# Borderline Ventricles: From Evaluation to Treatment

**DOI:** 10.3390/diagnostics14080823

**Published:** 2024-04-16

**Authors:** Giuseppe Antonio Mazza, Lilia Oreto, Giulia Tuo, Domenico Sirico, Sara Moscatelli, Giovanni Meliota, Antonio Micari, Paolo Guccione, Gabriele Rinelli, Silvia Favilli

**Affiliations:** 1 Division of Pediatric Cardiology, City of Health and Science University Hospital, 10126 Turin, Italy; 2 Department of Clinical and Experimental Medicine, University of Messina, 98122 Messina, Italy; 3 Pediatric Cardiology and Cardiac Surgery Unit, Surgery Department, IRCSS Istituto Giannina Gaslini, 16147 Genoa, Italy; 4 Pediatric Cardiology Unit, Department of Women’s and Children’s Health, University of Padua, 35128 Padua, Italy; 5 Centre for Inherited Cardiovascular Diseases, Great Ormond Street Hospital, London WC1N 3JH, UK; 6 Instutute of Cardiovascular Sciences, University College London, London WC1E 6DD, UK; 7 Pediatric Cardiology, Giovanni XXIII Pediatric Hospital, 70126 Bari, Italy; 8 Department of Biomedical, Dental Sciences and Morphological and Functional Images, Interventional Cardiology, University of Messina, 98122 Messina, Italy; 9 Mediterranean Pediatric Cardiology Center, Bambino Gesù Children Hospital, 98039 Taormina, Italy; 10 Pediatric Cardiology and Cardiac Arrhythmias and Syncope Unit, Bambino Gesù Children’s Hospital, 00146 Rome, Italy; 11 Department of Pediatric Cardiology, Meyer Hospital, 50139 Florence, Italy

**Keywords:** borderline left ventricle, borderline right ventricle, univentricular heart, one and half ventricle, CHD

## Abstract

A heart with a borderline ventricle refers to a situation where there is uncertainty about whether the left or right underdeveloped ventricle can effectively support the systemic or pulmonary circulation with appropriate filling pressures and sufficient physiological reserve. Pediatric cardiologists often deal with congenital heart diseases (CHDs) associated with various degrees of hypoplasia of the left or right ventricles. To date, no specific guidelines exist, and surgical management may be extremely variable in different centers and sometimes even in the same center at different times. Thus, the choice between the single-ventricle or biventricular approach is always controversial. The aim of this review is to better define when “small is too small and large is large enough” in order to help clinicians make the decision that could potentially affect the patient’s entire life.

## 1. Introduction

A heart with a borderline ventricle refers to a situation where there is uncertainty about whether the left or right underdeveloped ventricle can effectively support the systemic or pulmonary circulation with appropriate filling pressures and sufficient physiological reserve [1,2].

From an embryogenic point of view, defects in the expression or function of some genes (i.e., HAND-1 and HAND-2) may play important roles in ventricular hypoplasia. Furthermore, reduced or abnormal flow dynamics within the developing ventricle (e.g., in primary defects of the outflow tract) largely contribute to ventricular underdevelopment [3].

The aim of this review is to understand when “small is too small and large is large enough” in order to help clinicians make the decision that will potentially affect the patient’s entire life: the type of correction.

## 2. Methods

For this narrative review, we performed research in the PubMed library for the keywords “borderline left ventricle”, “borderline right ventricle”, “borderline ventricles echocardiographic scores”, “borderline left ventricle correction”, “borderline right ventricle correction”, “single ventricle follow-up” and “one and half ventricle follow-up”. The titles and abstracts of the articles published until October 2023 were all evaluated. Those written in languages other than English were excluded.

## 3. Borderline Left Ventricle: Predictors of Biventricular Repair

The concept of borderline left ventricle (LV) refers to part of a spectrum of cardiac diseases characterized by a small, underdeveloped LV [3]. The extremities of this spectrum usually do not pose any doubt regarding surgical treatment; severe hypoplasia, as determined by the mitral valve (MV) and/or aortic valve (AoV) atresia, needs single-ventricle palliation, whereas mild hypoplasia, such as in case of isolated aortic coarctation, generally requires biventricular repair (2VR). But the “grey zone”, which contains various forms of borderline LV, is always a matter of debate. In fact, the surgical management of these cases may be extremely variable from different centers, and the choice between the single-ventricle or two-ventricle approach is always controversial.

The first consideration in the case of borderline LV is that the heart team is required to make a decision nearly immediately after birth, which is different from the borderline right ventricle (RV), which allows for a longer watch-and-wait strategy as long as a source of pulmonary blood flow is provided.

Then, the early dichotomic choice taken in the neonatal age carries profound long-term consequences. Single-ventricle palliation consists of at least three-staged surgical operations (Norwood stage I, Glenn, and Fontan operation); on the other hand, 2VR is not necessarily free from complications and reinterventions, with a significant risk of diastolic dysfunction and pulmonary hypertension.

Conversion from one pathway to another is possible but carries high risks in both cases [4].

**Figure 1:**
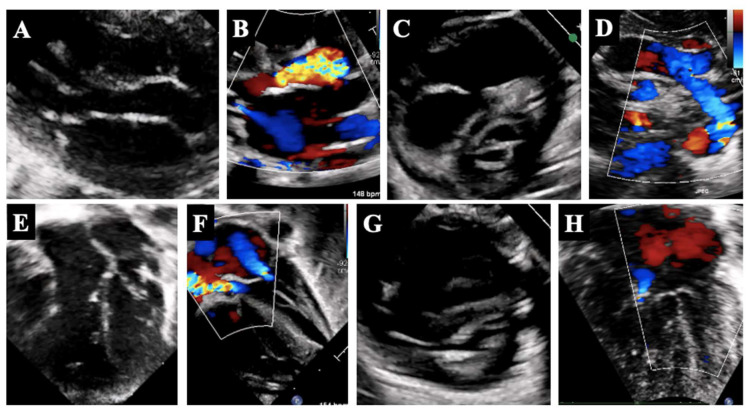
Variants of borderline LV. Panels (**A**,**E**) show mildly hypoplastic annulus of the AoV and MV without evident dysplasia of the valves; panels (**B**,**F**) show severe AoV stenosis with moderate MV regurgitation; panels (**C**,**G**) show hypoplastic MV with small opening, thickened leaflets and bright posterior papillary muscle; panel (**D**) shows hypoplastic transverse aortic arch; panel (**H**) shows unbalanced transitional AVSD with small left valvular size.

The anomalies associated with borderline LV include AoV stenosis, MV disease, and aortic arch hypoplasia, which can be variably combined. Also, an unbalanced atrio-ventricular septal defect (uAVSD) with right dominance produces a small LV (Figure 1).

The most characterized form of borderline LV, with a certain amount of literature available supporting the diagnostic definition of this disease, is critical AoV stenosis [5]. Even if the disease involves mainly the AoV, the MV and aortic arch may also be structurally abnormal, and the LV may show variable degrees of endocardial fibroelastosis (EFE), which represents the anatomical marker of a structurally altered LV.

Sometimes, anomalies of the MV may not be clearly evident at birth since the flow gradient across the valve may be absent in the first weeks of life; therefore, anatomic details of the valve should be considered, like the presence of a single papillary muscle or a dilated coronary sinus with persistent left superior vena cava, which may somewhat reduce the mitral inflow [6].

Although many studies over time have assessed the suitability of borderline LV for systemic circulation, there are no definitive rules available, and the issue is still open [2].

Echocardiography (ECHO) is certainly the main technique used, and historically, three major scores have been validated as risk prediction models for the 2VR:

- Rhodes score (1991) [5]:

14.0 (body surface area (BSA)) + 0.943 (indexed aortic root dimension) + 4.78 (LV to heart long axis ratio) + 0.157 (indexed MV area) − 12.03;

A mathematical multivariate equation that was obtained from 65 cases of critical aortic stenosis based on the measurement of the aortic root, MV area, and heart long axis. A score of less than −0.35 predicted death after 2VR in 88% of patients, retrospectively.

- CHSS equation (2001) [7]:

Survival benefit = Intercept + b1 (age at entry) + b2 (z-score of AoV at the sinuses) + b3 (grade of EFE) + b4 (ascending aorta diameter) + b5 (presence of moderate or severe tricuspid regurgitation (TR)) + b6 (z-score of the left ventricular length);

The Congenital Heart Surgeon Society’s multiple linear regression equation, validated in the case of multilevel obstruction of the left ventricular outflow, is based on the measurement of the aortic root, LV long axis, and also parameters not related to left heart valves, like ascending aorta, TR, EFE, and age.

The result gives the predicted difference in the percentage of survival for Norwood minus 2VR. A positive number would, therefore, favor a Norwood procedure, and a negative number would favor a 2VR.

- Discriminant score (2006) [8]:

10.98 (BSA) + 0.56 (aortic annulus z-score) + 5.89 (LV to heart long axis ratio) −0.79 (grade 2 or 3 EFE) −6.78;

A scoring system for the aortic stenosis in the presence of a normal MV, that is based on aortic annulus, the LV to heart long axis ratio, and EFE; a cutoff value of −0.65 accurately predicted the outcome in 90% of cases.

Although these scores are relatively easy to use, with online calculators freely available, there are significant limitations to their applicability in clinical practice.

First of all, there is no uniformity between the methods used in the different studies [9]; though the measurements required are simple, linear, and apparently easily reproducible, there is high variability in the way they are calculated. For example, the measurement of the aortic root can be obtained following the inner-to-inner edge or the leading-to-leading edge, in systole or in diastole. The z-score of the aortic root can be calculated with many different references; the BSA itself can be obtained with various methods.

**Figure 2:**
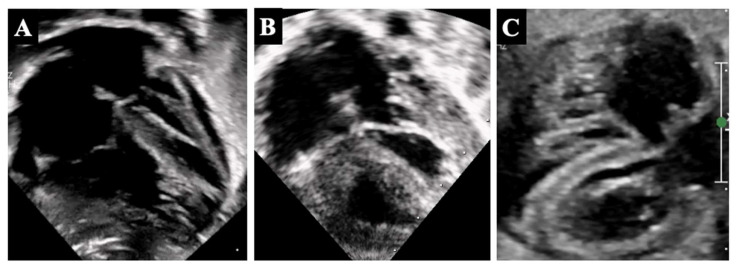
Panel (**A**) shows grade 1 EFE partially involving the sub valvular mitral apparatus; panel (**B**) shows grade 2 EFE with some endocardial border involved; panel (**C**) shows grade 3 EFE with involvement of the entire endocardial border and left ventricular remodeling.

Furthermore, for non-linear parameters like EFE, the evaluation is even more complicated. EFE is expressed as a semi-quantitative evaluation (grade 0–3), where 0 is absent, 1 represents the involvement of the papillary muscles only, and 3 represents the involvement of the entire endocardial border (Figure 2); however, its estimation is far from being reliable, since there are significant differences in the evaluation obtained by ECHO compared to that based on magnetic resonance imaging (MRI), and from pathology after surgery [10]. It should be noted that EFE is an important component of both the CHSS equation and the Discriminant score since it is strictly related to diastolic dysfunction. Thus, dimensions and volumes are important, but the growth potential of the ventricle and its function are profoundly influenced by the presence of EFE. MRI certainly has many advantages over ECHO in terms of the quantification of EFE as well as estimation of volumes, particularly in the case of uAVSD, but in the neonatal age, it requires dedicated radiologic settings and general anesthesia.

Furthermore, as frequently occurs in pediatric cardiology, also in this field of risk prediction models, most of the available literature consists of single-center, retrospective, and heterogeneous studies, with limited data regarding the follow-up, outcome, and functional status of the patients.

Finally, another limitation is that the Rhodes score and Discriminant score are validated exclusively for AoV stenosis, and CHSS for multiple left heart obstructions, therefore they cannot be used to evaluate borderline LV in the case of isolated aortic arch hypoplasia.

Studies evaluating scoring systems that are able to predict 2VR in neonates with aortic arch hypoplasia are available, based on the measurement of the MV, tricuspid valve (TV) and AoV annuli, left and right ventricular length, and main pulmonary artery diameter [11,12].

However, borderline LV in the presence of small but normally structured valvular components is usually expected to grow significantly even from presurgical, very low, end-diastolic volumes [2].

In fact, in these situations, known as the “hypoplastic left heart complex” (HLHC) and including the aortic arch hypoplasia and isolated coarctation of the aorta, left heart valves can be hypoplastic but without intrinsic stenosis or atresia, and the small LV is expected to recover as soon as the obstruction is removed [13].

In the case of uAVSD with right dominance, different criteria should be used to evaluate the borderline LV. First of all, the atrioventricular valve index (AVVI) has been proposed. From an “en face” image of the AV valve in the subcostal left anterior oblique view, the relationship between the AV valve and the ventricular septum is well displayed. The orifice of the common AV valve is traced in diastole. The left and right components of the common AV valve are generated by subtending the traced orifice along a line that corresponds to the intersection with the plane containing the interventricular septum. The left AV valve area is then divided by the total AV valve area to determine the AVVI, which ranges between 0.0 and 1.0. Right dominant hearts are defined as having an AVVI ≤ 0.4 and left dominant hearts as having an AVVI ≥ 0.6 [14]. The borderline LV has been proposed to have a ratio between 0.4 and 0.2, and in these cases, different ECHO parameters have been tested to predict favorable 2VR [15,16]. For example, the left ventricular inflow index (ratio between color-Doppler and anatomic left inflow diameter from the 4-chamber view) accounts for the amount of blood effectively preloading the LV; in addition, the right-to-left ventricle inflow angle (the angle formed between the virtual annuli of the left and right components of the common valve, traced from the base of the RV and LV free wall to the crest of the ventricular septum, from the 4-chamber view) expresses the entity of imbalance, with a steeper angle suggesting a more hypoplastic LV.

## 4. Borderline Right Ventricle: Predictors of Biventricular Repair

The definition of the borderline RV includes a wide spectrum of complex CHDs with neonatal presentation, such as pulmonary atresia (PA) with intact ventricular septum (IVS), critical pulmonary stenosis (CPS), severe Ebstein’s anomaly and uAVSD with left dominance [17].

In contrast to borderline LV, achieving a 2VR in newborns with borderline RV is not an impelling decision because additional sources of pulmonary blood flow can be provided by surgical or catheter-directed methods [18]. In fact, patients with inadequate oxygen saturations after neonatal right ventricle outflow tract (RVOT) decompression may be treated with a surgical shunt or ductal stent, possibly with a plan for 2VR or ventricular recruitment procedures later in life.

At present, the patient selection and timing of 2VR likely represent the major determinants of the outcome. Many measurements have been used to define the borderline RV and to assess the potential candidacy for 2VR. ECHO and/or MRI may provide quantitative measurements as z-score of TV size, the TV cross-sectional area, RV end-diastolic volume and stroke volume. Cardiac catheterization allows the collection of physiologic data, specifically the right atrial pressure, right end-diastolic pressure, and cardiac output. Unfortunately, none of them represents the gold standard for predicting whether the size and performance of a borderline RV are adequate to support pulmonary circulation [19,20,21].

During the neonatal period, ECHO is still the main imaging modality used in the diagnosis and estimation of disease severity, which influences surgical/interventional planning in neonatal CHDs with borderline RV. Several studies tried to evaluate which echocardiographic measures were able to predict the outcomes, including successful 2VR.

Nevertheless, the echocardiographic parameters and scores for disease severity and risk prediction proposed until now have not been homogeneous [22].

Most of the literature is focused on PA-IVS or CPS and borderline RV.

PA-IVS is defined by the lack of communication between the RV and the pulmonary artery. It is characterized by a heterogeneous morphology, especially of the RV, various degrees of TV dysplasia, and coronary anomalies. Therefore, treatment algorithms and surgical management decisions need to be tailored to individual morphologic subtypes at presentation [23]. The main goal in the newborn period is to predict the suitability for long-term biventricular, univentricular (completed Fontan operation with or without fenestration), or “one and half” ventricular repair. Fundamental to these decisions are the following points:

- The determination of whether the RV can support (or can be rehabilitated to support) a full pulmonary circulation and the systemic venous return;
- The morphologic assessment of the RV (unipartite, bipartite, or tripartite) and the infundibulum (membranous vs. muscular pulmonary atresia);
- The assessment of the TV size, morphology, and function;
- The presence of coronary sinusoids and of right ventricular dependent coronary circulation (RVDCC) [24,25,26].

For patients with mild-to-moderate hypoplasia of the RV who generally have a patent infundibulum, the treatment goal is to establish a 2VR with RV decompression as the initial and often unique procedure. Patients with a severely hypoplastic RV who often have major coronary anomalies with RVDCC are generally assigned to univentricular repair. The most challenging case is the PA-IVS with moderate-to-severe hypoplasia or borderline RV, that usually shows a tricuspid annulus z-score from −2.5 to −4.5, bipartite RV (an absent or markedly attenuated trabecular component), commonly small pulmonary valve (PV) annulus with or without membranous atresia and/or sub valvular stenosis, and commonly minor sinusoids without RVDCC.

Many reports have investigated the optimal initial management strategy for this scenario using various morphologic findings at presentation as predictors. However, the best morphological features, which may be prerequisites for 2VR, are not yet known.

One of the largest studies led by the CHSS proposed a surgical management algorithm dependent on the TV z-score at presentation, considering that the TV annulus is easily measured and directly reflects the RV size [27]. A total of 171 neonates with PA-IVS were entered into a prospective study from 31 institutions between 1987 and 1991. A surgical pulmonary valvotomy without a concomitant shunt appeared optimal in neonates in which the z-value of the TV diameter was −0.15 or larger. In those in which the z-value was between −1.5 and −4, the optimal initial intervention was the insertion of a transannular patch and, concomitantly, the placement of a systemic-to-pulmonary artery shunt. The most arguable issue remained the management of patients with normal or near-normal coronary circulation but with very small TV (z-values less than −4). In such patients, the risk of an initial procedure of concomitant transannular patching and systemic-to-pulmonary artery shunting appeared to be high, but such a procedure was deemed to offer the possibility of the subsequent enlargement of the TV and, ultimately, a 2VR.

Since this report, an additional 237 neonates were prospectively enrolled, and the new cohort included all 408 neonates with PA-IVS admitted within 30 days after birth to a CHSS institution from January 1987 to April 1997. They demonstrated that both the TV z-score and RV size were important determinants for achieving a 2VR, either early or late. In addition to TV size, TV morphology proved to be an important factor determining the suitability for 2VR [28].

Throughout the ages, several other reports were published, but the minimal TV z-score needed to predict a successful 2VR remained controversial, with values ranging from −2.4 to −4 [27,29,30]. A disadvantage of z-scores is that they depend on the distribution of normal values in a specific population, and if the data are not available, widespread generalization may not be appropriate.

Minich et al. in 2000, demonstrated the usefulness of the preoperative TV/MV ratio for predicting outcomes in PA-IVS. A ratio > 0.5 was the best predictor of a 2VR, requiring no statistically generated information and allowing each infant to provide his or her own normal data. When *p*-values were compared, a ratio > 0.5 was a better predictor of a successful 2VR than a tricuspid valve z-score > −3 [31].

Drighil et al. in 2010, identified specific echocardiographic markers that were predictive of success for radiofrequency perforation and balloon pulmonary valvuloplasty (RFV) in those patients with membranous-type PA–IVS. According to them, an RV/LV diameter ratio > 0.76 predicts a 92.3% success rate. In contrast, an RV/LV diameter ratio ≤ 0.76 associated with an RV/LV length ratio ≤ 0.70 predicts 100% failure. [32].

In 2017, the Congenital Catheterization Research Collaborative (CCRC), made up of four large pediatric centers (Children’s Healthcare of Atlanta, Children’s Hospital of Philadelphia, Cincinnati Children’s Hospital Medical Center, and Texas Children’s Hospital), aimed to identify widely applicable risk factors for reintervention and failure to accomplish 2VR through a retrospective analysis on a large multicenter cohort of PA-IVS patients, whose treatment strategies were comparable [33]. The final study cohort consisted of 99 patients who received an intervention during the neonatal period (age ≤ 30 days). The authors found that outcomes after decompression were more strongly associated with the degree of TR than with the TV z-score and other right heart parameters, as reported in the past. Lesser TR was a predictor of worse outcomes, with a greater incidence of the need for additional pulmonary blood flow after RV decompression. It is reasonable to link the pre-intervention degree of TR with the development of the RV and its parts, both in fetal and post-natal lives, in the setting of PA-IVS. The degree of TR, in fact, correlates directly to the RV inflow volume, because the blood volume entering the RV cavity before decompression is equal to the regurgitant volume at the TV (excepting the RV-to-coronary flow in neonates with fistulous connections). A higher degree of TR was associated with a better TV annulus diameter, TV annulus z-score, and inflow duration.

Afterward, the CCRC performed a new multicenter, retrospective study that included the same study population of all infants with PA-IVS who underwent RV decompression at less than 30 days of age between 2005 and 2015, with the aim of better clarifying the characteristics of RV which are ultimately sufficient for 2VR. Specifically, the authors’ objectives were to identify baseline and postdecompression echocardiographic parameters associated with RV growth and successful 2VR. ECHOs were analyzed at the following three time points: the baseline (preintervention), postdecompression (first postintervention ECHO), and at follow-up (closest to 1-year postdecompression, or last ECHO prior to Glenn operation). The TV annular dimension and RV area were measured during diastole on the baseline and follow-up ECHOs. They found that a baseline RV area ≥ 6 cmq/mq had a high discriminative value in identifying patients who achieved 2VR. Preintervention parameters associated with greater RV growth included a larger TV annulus z-score and ≥moderate TR. Postdecompression parameters associated with achieving a 2VR included a ≥moderate TR and higher PV velocity. During the follow-up, an RV area ≥ 8 cmq/mq at 1 year appeared to be a useful predictor for successful 2VR. A larger baseline TV z-score, a ≥moderate baseline TR, and ≥moderate postdecompression pulmonary regurgitation (PR) were all associated with the achievement of this threshold [34].

Furthermore, Chen R.H.S. et al. in 2018, focused on the outcome of newborns with moderate hypoplastic RV (mean TV z-score −4.2 ± 3.0, 69.4% of patients with z-score < −2.5) who underwent RFV even if they presented an unfavorable form of PA-IVS [35]. The study represented a series with one of the longest postprocedural follow-ups reported for patients with PA-IVS managed with RFV (median 10.7 years). They demonstrated that a TV/MV annular dimension ratio of more than 0.79 could confidently predict a successful 2VR (specificity and positive predictive value of 100%). Compared to the TV/MV annular dimension ratio cutoff of 0.5 previously suggested by Minich et al., this new cutoff presents better specificity.

Recently, Giordano et al. reported their single-center study, which analyzed 62 consecutive neonates with PA-IVS or CPS referred to the Monaldi Hospital, Naples, from January 2010 to September 2021 [36]. The main aim of the authors was to find predictive markers of persistent duct dependency of pulmonary circulation (PDDPC) after successful RV decompression. They analyzed the clinical, hemodynamic, and echocardiographic data of all neonates who underwent percutaneous treatment from 2010 to 2021, and they built the PDDPC score. The variables included were the TV z-score, TV/MV annular ratio, PV z-score, end-diastolic RV area, end-systolic right atrium area, the percentage amount of interatrial right-to-left shunt, TR, RV systolic pressure, and E/E′ ratio. These nine echocardiographic parameters were arbitrarily assigned +1 point each when they exceeded the respective cut-off value. Thus, the PDDPC score ranges from 0 to 9 points. A score cut-off ≥4 seems able to predict PDDPC with both high sensitivity (100%) and specificity (86%), allowing to better plan the correction strategy.

## 5. Surgical Strategies

In cases of borderline ventricles, alternative surgical strategies have been pursued over the last decade.

### 5.1. Borderline Left Ventricle

Borderline LV, by definition, always has growth potential related to the amount of blood flowing through it, given the equation “flow = grow” [37].

Neonatal 2VR has been reported to be feasible in a population with specific characteristics by Tchervenkov et al. [38], including patients with hypoplasia of all the structures within the left heart–aorta complex without intrinsic AoV or MV stenosis (HLHC).

For those cases that remain in the “grey zone”, after an initial single-ventricle palliation, the “staged LV recruitment” has been proposed to volume load the LV and promote its growth over several months to years. It includes AoV and MV surgery, the resection of EFE, atrial septal defect restriction, and the augmentation of pulmonary blood flow by a modified BT-shunt or Sano conduit in addition to bidirectional Glenn (BDG) [39,40,41,42].

Nonetheless, only one-third of the patients undergoing staged LV recruitment are reported to reach 2VR [43].

There are also some experiences showing positive outcomes for delayed 2VR in borderline LV with mild hypoplasia after the neonatal hybrid palliation. In fact, this procedure may allow postponing the decision between single-ventricle palliation or 2VR until the LV has expressed its growth potential, usually after 4–6 months [44,45,46].

**Figure 3:**
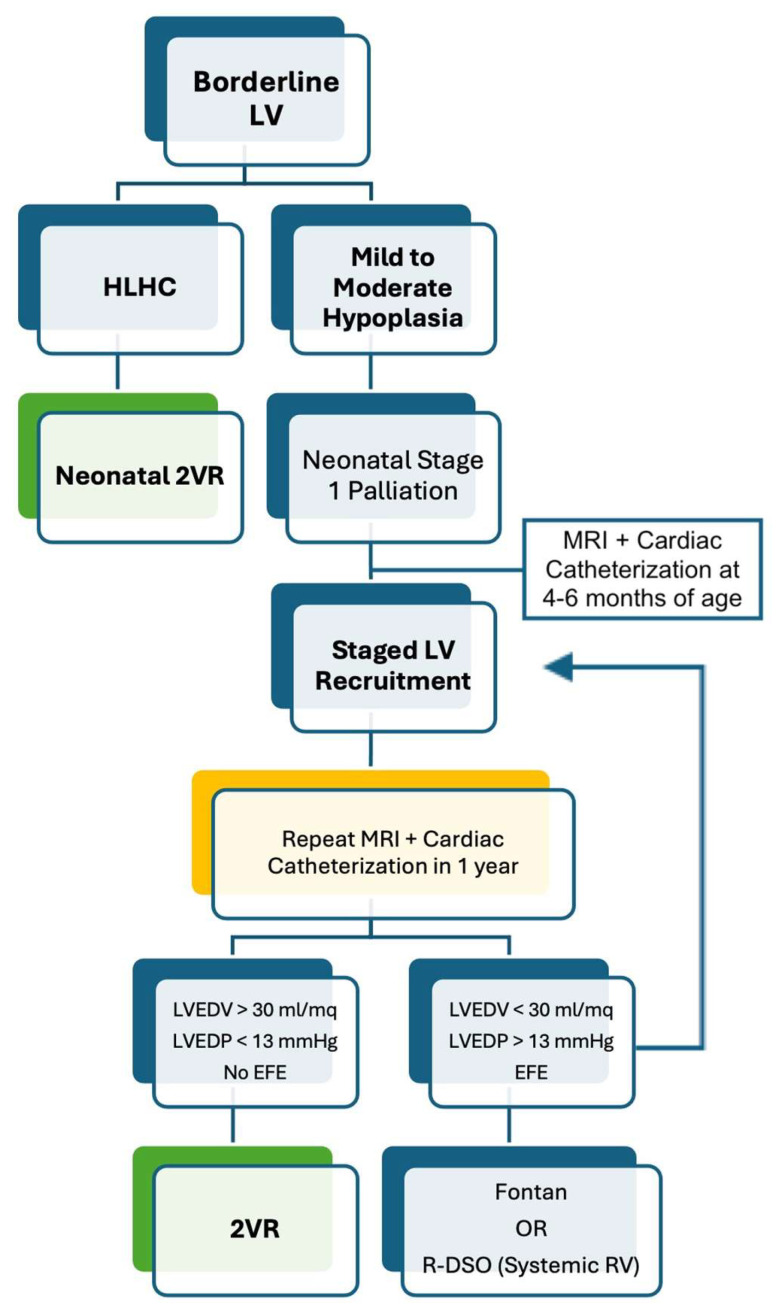
Algorithm for the treatment of borderline LV. LV: left ventricle; HLHC: hypoplastic left heart complex; 2VR: two ventricular repair; LVEDV: left ventricle end-diastolic volume; LVEDP: left ventricle end-diastolic pressure; EFE: endocardial fibroelastosis; R-DSO: reverse double switch operation.

A possible new strategy, the “reverse” double switch operation (R-DSO), has been recently described by Scully et al. [47]. This procedure includes atrial and arterial switch to utilize the borderline LV as the sub-pulmonary ventricle and the RV to support systemic circulation. Further unloading of the LV can be achieved with a BDG anastomosis or a fenestrated interatrial septum. Such a strategy was applied by the team in Boston to patients with borderline LV who either failed LV recruitment for 2VR or presented risk factors for Fontan completion (e.g., pulmonary hypertension, pulmonary vein stenosis). The advantages rely on low inferior vena cava pressures and possible recovery from pulmonary arterial hypertension [48]. However, long-term follow-up is necessary to determine the duration over time of this strategy (Figure 3).

### 5.2. Borderline Right Ventricle

Patients with functionally or anatomically borderline RV may present a higher risk of complications and mortality after 2VR [49]. Predictors for 2VR have been exposed.

In PA-IVS or CPS, early borderline RV decompression can promote both TV and RV growth through improved antegrade pulmonary blood flow.

Patients with inadequate oxygen saturation following RVOT decompression may be treated with a surgical shunt or ductal stent in the neonatal period, with a plan for ventricular recruitment procedures like transannular patch, and possibly biventricular conversion afterwards [27,29,50,51,52].

For those cases that remain in the “grey zone”, an alternative strategy to the Fontan palliation is the “one and half ventricle repair” (1.5VR). This term was introduced by Billingsley et al. in 1989 for the treatment of a subgroup of patients with PA-IVS and moderate RV hypoplasia who underwent BDG anastomosis at the time of the 2VR [53]. The principle beyond 1.5VR is the off-loading of the RV through the BDG, which reduces the preload by approximately one-third [54]. The decision to undergo 1.5VR rather than 2VR or Fontan palliation relies on the assessment of the sub-pulmonary ventricle regarding its capability to support pulmonary circulation without failing and without a significant rise in systemic venous pressures. Important factors to evaluate include the TV size z-score, RV size, RV compliance, TR, the morphology of the RVOT, degree of pulmonary artery hypoplasia, and pulmonary vascular resistance (PVR).

The selection criteria proposed by Talwar S. and al. are a TV z-score between −4.8 and −1.5, a TV diameter between 45 and 70.5% of normal, a TV/MV ratio of between 0.54 and 0.80, and RV diastolic volumes ranging between 45 and 75% of the predicted normal volumes [55].

Absolute contraindications to 1.5VR are represented by RV-coronary artery fistulas and RVDCC. In fact, in these cases, RV decompression may precipitate fatal coronary ischemia. In addition, pulmonary arterial hypertension and complex intracardiac repair with prolonged cardiopulmonary bypass (CPB) time may represent relative contraindications to this approach. The 1.5VR showed good early (90–96%) and late (80–90%) survival. Furthermore, in a study by Kim et al., among the survivors, 98.8% of patients were in NYHA class I or II [56]. Finally, Cabrelle et al. reported that freedom from any late adverse event was 57.1%, and freedom from reoperation and interventional procedures was 82.1% and 78.6%, respectively, with no significant difference in survival and in freedom from adverse events between simple and complex anatomy groups, in a long-term follow-up [57].

**Figure 4:**
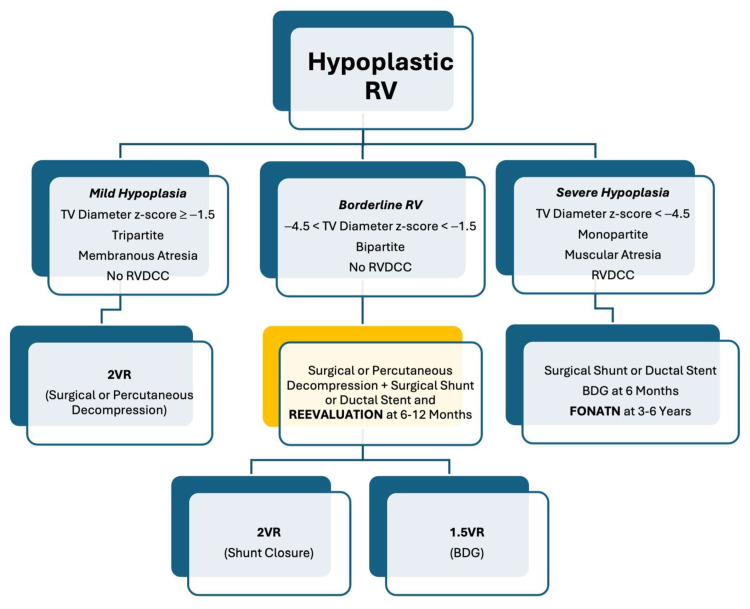
Algorithm for the evaluation and treatment of the hypoplastic RV in PA-IVS. RV: right ventricle; TV: tricuspid valve; RVDCC: right ventricular dependent coronary circulation; 2VR: two ventricular repair; 1.5VR: one and half ventricle repair; BDG: bidirectional Glenn. The values of TV diameter z-score are indicative only.

In conclusion, the 1.5VR represents a valid alternative to Fontan palliation in a subgroup of patients with borderline RV, considered at high risk for 2VR (Figure 4). Furthermore, it may serve as a rescue strategy for patients with small RV and failing 2VR.

## 6. Post-Surgery Period

In cases of surgical 2VR, the borderline ventricle often exhibits signs of diastolic dysfunction and non-compliant physiology in the early postoperative period. To manage this, the direct measurement of right and left atrial pressures, administration of inotropes, and temporary paralysis are utilized for approximately 3–4 days following surgery. As ventricular physiology normalizes, the level of support is gradually reduced. Additionally, an atrial fenestration is commonly left in place to prevent severe left or right atrial hypertension during this early period. Another common occurrence during the first 72 h after surgery is the presence of fever. The exact cause is uncertain, but it may be associated with a low cardiac output state, a systemic inflammatory response triggered by the surgical procedure, or changes in circulation. To prevent hemodynamic instability, fever is actively managed through the use of antipyretics and localized cooling measures. Also, pulmonary dysfunction, characterized by severe pulmonary edema and/or atelectasis, is frequently observed, potentially resulting from factors such as CPB, transfusion reactions, or reperfusion injury during the transition from passive to pulsatile pulmonary blood flow. Extended periods of ventilation, diuresis, and transitioning to positive pressure ventilation are often necessary to address this issue [17]. A systemic inflammatory response that occurs after CPB surgery significantly contributes to the aforementioned problems and may also lead to dysfunction in other organs. Since the biventricular conversion procedure, especially for the borderline LV, often requires prolonged CPB times, a subsequent prolonged intensive care unit (ICU) stay is generally needed [58].

In the case of 1.5VR for borderline RV, postoperative complications can include the following: superior vena cava dilation with elevated pressure, and the subsequent increased incidence of chylotorax and pleural effusion early after the operation. Later, patients can develop significant PR, which can lead to a semi-circular shunt from the superior vena cava, increasing the sub-pulmonary ventricle preload and nullifying the benefits of the repair [57].

Patients with univentricular repair, as well expressed by the “ticking clock theory”, commonly have a limited lifespan. This is because palliation with total cavopulmonary connection exposes to significant complications, particularly the risk of ventricular failure. Identifying predictors of ventricular failure allows us to monitor and possibly intervene, through percutaneous or surgical methods, to delay its worsening [59,60]. The primary underlying causes of Fontan failure include the regurgitation of atrioventricular and semilunar valves, chronotropic insufficiency, the burden of systemic-to-pulmonary collaterals, myocardial and hepatic fibrosis.

## 7. Conclusions

Pediatric cardiologists often deal with CHDs associated with various degrees of hypoplasia of the left or right ventricles.

To date, no specific guidelines exist for borderline cases, and surgical management may be extremely variable in different centers and sometimes even in the same center at different times. Thus, the choice between the single-ventricle or 2VR approach is always controversial.

With this review, we try to give a supplemental tool to clinicians in order to choose the best treatment for every single patient.
